# Metabolic agricultural ethics: Violence and care beyond the gate

**DOI:** 10.1177/27539687231155224

**Published:** 2023-02-09

**Authors:** George Cusworth

**Affiliations:** 6396 University of Oxford, UK

**Keywords:** Agricultural ethics of care, anthropocene, sustainable food, agriculture, care, feminist ethics

## Abstract

A substantial body of scholarship now exists describing an agricultural ethics of care. This work has integrated insight from feminist ethicists into research on food production, human–nature relations, and agricultural land use. As scholars elsewhere in the humanities have discussed, though, there is often a violence committed in care's name. In the case of food production and farming, I argue that the focus on affect, local multispecies relations and a proximal encounter-based ethics risks obscuring ethically significant and potentially violent food system dynamics that unfold beyond the farm gate. To better accommodate these remote yet important outcomes, I argue that scholars deploying an ethics of agricultural care should pay greater attention to the metabolisms of the farms, labs, nurseries, gardens, and allotments they study. Such an approach can accommodate those things that enter the case study site (fertilizer, animal feed, seed, etc.) and those things that leave it (vegetables, grain, pollution, etc.) as well the more-than-human transformations and interactions that take place within it. By being attentive to these material inflows and outflows, new ethical responsibilities emerge to act in the speculative hope that violence can be minimized, and care can flourish across a broader spatial range.

## I Introduction

The idea of care has come to occupy an important part in contemporary social science thinking on food and farming. Building on Tronto's (1993) feminist scholarship highlighting the gulf that exists between the political potential of care, and the cultural denigration of those (mostly women) who administer it, this research has investigated how an ethics of care can help navigate the various environmental, social, and animal welfare problems in the food system (Puig de la Bellacasa 2015). Food production is reliant on the wellbeing of a wide variety of bugs, bacteria, fungi, plants, critters, soils, etc., and researchers have invoked critiques of nature–culture dualisms (Braun 2009; Whatmore 2002) to accommodate agricultural multispecies entanglements in the enactment of care. The agricultural care framework that has emerged is one centered on a relational, post-humanist ontology, and a proximal encounter-based ethics. Guided by affect, sense, and empathy, this work has called for food production practices that are more caring and attentive to the multispecies worlds that constitute agro-ecosystems (Carolan 2015).

Care, though, has a “darker side” and there is often a “violence committed in its name.” (Martin et al. 2015, 627). Martin et al. (2015) preface their Special Issue on care by asking *cui bono (who stands to gain)?* Care is not, they show, an unqualified and universal good, and work needs to be done to understand the politics involved in defining who benefits from an ethics of care, who decides how care is to be enacted, and who has to undertake the caring labor. The notion of violence (both within and beyond the Special Issue Martin and colleagues introduce) figures as the antithesis to care: it is used to draw attention to the ways in which the enactment of care can visit harms on some or all of the actants implicated in a given assemblage (van Dooren 2016; Giraud et al. 2019); the way the distribution of care's benefits and responsibilities are indexed to pre-existing power hierarchies relating to race, gender and species (Murphy 2015; Srinivasan 2013); and the way the enactment of care forecloses on the possibility of other worlds in which the benefits and costs of care would have been distributed differently (Giraud 2019). Violence is, in other words, an ever-present accompaniment to care: it is the cost of the care's labor, and the unavoidable upshot of enacting care for the benefit of some actors and not others. The utility of invoking violence in this way is derived from the same “slipperiness” (Martin et al. 2015) that has allowed care ethics to become so powerful and popular. It is context-specific, messy and malleable.

The paper's overarching aim is to think through the implications these concerns have for the scholarship applying care to food production contexts. What violence, in other words, might be being committed in the name of agricultural care? Like Martin et al. (2015) do in their introduction, I am asking this question not to itemize the specific harms generated through specific articulations of care, but to initiate thinking on how care's tendencies for particularism might be known and, in turn, lessened. I am also asking the question in the most generative and collaborative spirit. Scholars have covered commendable ground applying care ethics to agricultural contexts, with real world applications for how food producers go about their work (Krzywoszynska 2020). What conceptual developments are needed, though, to broaden agri-care's ethical coverage and to minimize the violence that care is always capable of producing?

I argue that agri-care scholarship's focus on proximal and affecting encounters and multispecies entanglements is obscuring the agro-ethical significance of remote and diffuse food system dynamics that unfold beyond the farm gate. Individual farmers managing individual farms are imbricated in the food system's global web, and decisions made in one location have consequences that ripple out and touch down in distant others (Beacham 2022; Yao et al. 2018). Food production operations are linked to the food system they operate within by those things that are brought onto (animal feed, labor, fertilizers, diesel, etc.) and leave (fruit, grain, greenhouse gas emissions, water pollution, etc.) the site in question. These material vectors are a vital part of their *metabolism* (Swyngedouw 2006) and they can, I believe, be used to gain analytical purchase on the outcomes that might otherwise get missed by an ethical framework predicated on affect, ontological entanglement and multispecies encounter. The inclusion of these metabolic inflows and outflows in a framework of agricultural care is not intended to displace or challenge the considerations already articulated in the literature, but to add to them.

To accommodate these additional considerations in the enactment of care, I build on central features of its ethical grammar. By being attentive to the material vectors rooting a given food production context to the broader system it operates within, and by learning to affected by all the research and knowledge revealing the ethically significant impacts these metabolic inflows and outflows produce elsewhere in the world, scholars can speculate about the violence and/or care unfolding beyond the farm gate. In presenting these suggestions, I am not implying that the scholars deploying notions of agricultural care in their work aren’t interested in these broader food system issues; nor that the farmers and producers at the heart of their studies aren’t already acting on them. The aim, instead, is to initiate thinking on the conceptual developments needed to ensure that notions of agricultural care can produce salutary outcomes both near and far from the sites being studied, and to minimize the violence created in its name.

The paper's contributions are twofold. The first is to review the now significant body of writing on care in agricultural and food production contexts. This review work can be found in the following section. The second aim is to petition agri-food scholars to pay greater attention to the metabolic inflows and outflows of their case study sites to ensure that the ethical currency assigned to local and affecting outcomes in agri-care frameworks does not come at the expense of its engagement with remote yet ethically significant outcomes. This can be found in Sections III, IV, and V. Section III focuses on the risk of particularism in care's current agricultural formulation; Section IV on the application of the concept of metabolism to agri-food research; and Section V on the (continued) role of attentiveness, affect and speculation in the model agricultural care ethics presented in this paper. In the paper's conclusion, Section VI, I situate this paper back in the feminist ethics literature from which the agricultural care project was launched. I show the resonances it has with long-running efforts to expand care's geographic coverage and to minimize its potential for violence.

## II An agricultural ethics of care

Puig de la Bellacasa's first writing on care in the context of food and farming (2010) has inspired a fair amount of empirical and theoretical work on the subject. There, she reviewed the feminist scholarship on care, both the work that has used it in sociological inquiries into the delivery of (mostly unpaid, mostly female) care work (Malos 1980) and as an ethico-political project (Jaggar 2001; Tronto 1993). Care, as this literature shows, has great merit once it is allowed to flourish outside the confines of the private realm to which it has historically been confined. Following Fisher and Tronto, care is understood to encompass “everything that we do to maintain, continue, and repair our ‘world’ so that we can live in it as well as possible” (1990: 40). In combining insights from feminist care literatures with scholarship on biopolitics and human–nature relations, Puig de la Bellacasa (2010) initiated the project of tracing out how a feminist ethics of care might be called on to navigate various environmental and social problems associated with the production of food. While care theory has been applied to agricultural contexts without these conceptual underpinnings (e.g., Harbers 2010), it is this post-humanist strand that has come to have most traction in agri-food social science. I describe the literature Puig de la Bellacasa's work helped precipitate under three subheadings: *relational ontology and everyday ethical doings; attentiveness and affect;* and *technology and time.* These have been selected to capture the theoretical construction and various empirical findings of the research on agricultural care ethics.

### 2.1 Relational ontology and everyday ethical doings

Starting with Puig de la Bellacasa's (2010) reading of the notion of *naturecultures* (Haraway 1997, 2008; Latour 1993) into the Permaculture movement, Haraway's relational ontology, in which “beings do not pre-exist their relatings” (Haraway 2003: 6), has become a central feature of the agricultural care literature (Beacham 2018; Krzywoszynska 2019a; Mincyte et al. 2020; Puig de la Bellacasa 2010, 2017). Per the erosion of ontological categories inspired by new materialist thinking, and per the distribution of agency to the various humans and nonhumans that make up the world, ethical obligations “are not limited to what we traditionally consider care relations: care of children, of the elderly, or other ‘dependants’” (Puig de la Bellacasa 2010: 164). They are, instead, expanded to include those various nonhumans that constitute the complex web of an agroecosystem (crops, soil, farmers, grass, fungi, birds, bees, rivers, livestock animals and so forth).

Care is thus based on “ontological rather than moral or epistemological” foundations (Puig de la Bellacasa 2017: 69). It is the function of the mutual ontological constitution of humans and nonhumans in agroecosystem-nature cultures, and the responsibilities this entanglement produces. This ontological move functions as a response to the violence produced through a speciesist prioritization in productivist farming systems of human over other-than-human needs (Beacham 2018; Puig de la Bellacasa 2015). Across the literature, care is pursued for the benefit of both the humans on and around the farm, as well as the agro-ecosystem of which the farm is composed (Mincyte et al. 2020; Moriggi et al. 2020; Sovová et al. 2021). Citing these diverse beneficiaries, Esteve Giraud (2021)^1^ argues that care in the context of food production can be called on to meet high-level political targets like the UN's Sustainable Development Goals.

In line with the feminist writing from which the literature draws, agricultural care ethics is indivisible from the enactment of everyday caring actions. Puig de la Bellacasa refers to these as *everyday ethical doings:* “a life sustaining activity, an everyday constraint” (Puig de la Bellacasa 2010: 164). Far from being isolated acts of moral laudability or confrontational political interventions, caring is a constant expression of an internalized way of relating to the more-than-human world and one's sense of duty towards its wellbeing. This quotidian aspect of care runs through the literature. Beacham (2018) senses it in the diplomatic reactions urban farmers offer for the deer that eat the farm's produce; Krzywoszynska (2019a) in the hopeful way farmers iteratively improve their sustainable soil practice; Morrow and Davies (2022) in the way volunteers produce compost for circular food economies; Sovová et al. (2021) in the act of providing nourishing food for the local community and for the insects and plants in “garden ecosystems”; Pigott (2021) in the way biodynamic farmers take time to imbue the water with atmospheric essence before applying it to the land; and Münster (2021) in the way cattle urine and dung fermentations are prepared and spread in Natural Farming systems. The enactment of care and the minimization of violence visited on other-than-human life have begun to enmesh with cultural preferences defining what it is to be a “good farmer” (Cusworth 2020; Franklin et al. 2021; Larder 2021).

Soil has emerged as a vital area of study in this literature: a perfect site for thinking through a framework of ethical care in an agricultural context. Soil is, according to the “hydroponic” Krzywoszynska (2019a) western agricultural paradigm, little more than the substrate in which food is grown (Kearnes and Rickards 2020). When knowledge produced through soil testing of agronomically pertinent metrics (water content, nutrient levels, friability) is paired with extensive use of synthetic nutrient inputs and pesticides, complex soil ecologies become of limited functional value. While this system has been able to underpin rapid advances in productivity, issues of erosion, loss of soil structure and ecology are now common across agricultural soils all over the world. An approach to soil that directs its caring attentions only to those metrics most straightforwardly indexed to crop growth thus visits a violence on soil as an ontological whole (O’Brien 2020).

To reverse the harms inherent in these dynamics, agri-care scholars have argued the status quo agro-epistemological paradigm must be challenged (Salazar et al. 2020). Soil care thus involves an ontological reimagination of the hydroponic framing that subtends the productivist agricultural regime just as much as it involves the adoption of the practices (like no-tillage) that are typically called on to improve soil health (Krzywoszynska et al. 2020). The practitioners that have proven themselves best equipped to care for their soils also recognize—even embrace—the speculative approach that must be adopted when caring for something whose wellbeing is governed by highly complex biophysical processes, and on to which it is difficult to project neat ontological categories (Krzywoszynska 2019a).

### 2.2 Attentiveness and affect

Attentiveness in care is often—although not exclusively (Harbers 2010)—bound up with indigenous and philosophical animism and requires people to avail of their senses in a way that can produce new intimacies and perceptions of the more-than-human world they inhabit (Bird Rose 2013). Being attentive in food production contexts is central to an actor's ability to understand the way that different management practices produce violent and/or caring outcomes for a farm's multispecies assemblage (Krzywoszynska 2019a). Attentiveness has been used to characterize the care manifest in the relations between alternative protein insect farmers tending their “mini-livestock” (Bear 2021); the attention to detail wine makers pay to the complex needs of vines in viticultural production (Alarcon et al. 2020; Krzywoszynska 2016); the maternal instincts nursery workers have for the seeds and whips in palm tree production (Chao 2018); and the way farmers navigate environmental regulations around nitrogen water pollution (Franklin et al. 2021). In regenerative farming systems, being attentive to the agency of agricultural multispecies entanglements can help farmers grapple with the needs of the specific landscapes they manage, and to avoid the violence that can be generated through the application of a standardized model of agricultural practice (Seymour and Connelly 2022).

Affect is as an important feature in conceptualizations of agricultural care, and the ways in which practitioners can become attentive to the needs of multispecies life (Carolan 2015). Building on the relational ethics literature, farmers must become *response-able* (Krzywoszynska 2019a) to the needs of the nonhuman others on the farm - or *learn to be affected by them* (Despret 2004; Van Dooren et al. 2016) as it is put elsewhere in the environmental humanities literature—before they can be attentive and caring. Affective moments precipitated by feelings of wonder, disgust, sympathy or affection can catalyze an “ethical contagion” (Yusoff 2013), in which the cohort of (nonhuman)actors to which people have a sense of ethical commitment is expanded. Carolan (2015, 2016) terms this the *affective* turn in agri-food scholarship.

The constituent components of agricultural care—ontological entanglement, affect, attentiveness, and everyday caring behaviors—exist in a self-sustaining rhythm (Alarcon et al. 2020; Leung and Darnhofer 2021; Moriggi et al. 2020; Puig de la Bellacasa 2010). The farmers who embrace their interconnected and mutual constitution of agro-ecosystems become attentive to and affected by the needs of their nonhuman co-habitants; and the enactment of caring ethical doings, guided by affect and attentiveness, further stimulates commitment to an ontological framing that emphasizes the indivisibility of human–nonhuman entanglement.

### 2.3 Time and technology

Scholars have identified the dissonance that exists between the temporality at work in soil health and repair (slow, governed by unclear biophysical processes) and the temporality that choreographs soil's role in productivist agricultural systems (organized around annual cash-crop production, reliant on tillage, and fertilizer applications) (Krzywoszynska 2019a; Puig de la Bellacasa 2015; Salazar et al. 2020). To mitigate against the violence this temporal mismatch can produce, there is a need for land managers to “make time” for soil (Krzywoszynska 2019a; Puig de la Bellacasa 2015). The injunction carries a dual meaning. On the one hand, it is about the need for farmers to give themselves the time to tune in, and be affected by the soil and its lively, complex behaviors. On the other, time-making refers to the ontological concessions farmers must make to the multiple timescales involved in soil repair.

For Morrow and Davies (2022), the time and care volunteers invest in an inner-city composting initiative is central to the commitment they feel towards the principles of a circular food economy. Considerations of “timeliness” also color the care farmers have for pollution levels in local waterways, and the attentiveness needed to grasp the passage of time that tethers some agricultural activities (like fertilizer application) with some potentially violent environmental outcome (like nutrient run-off) (Franklin et al. 2021). In Mincyte et al. (2020) study of post-Soviet urban allotments, time is used to frame the life-histories of different cohorts of allotmenteers (young, looking to unwind; older, interested in self-provisioning) and the impacts this has on how they conceptualize their caring duties.

The idea of *technology* also cuts the agri-care literature. Werkheiser (2018) discusses the immiscibility of caring relations and precision livestock technologies. For him, everyday caring activities become harder for farmers to enact when they are kept at arm's length from the livestock animals by ag-tech developments like animal facial recognition and robotic milking infrastructures. By mediating farmers’ engagement with the livestock, there is a violence implicit in the way these technologies frustrate farmers’ ability to be attentive to the characteristics and needs of individual animals. Graddy-Lovelace (2020) discusses the dwindling room being left for traditional seed-saving and crop breeding practices by industrial breeding programmes. She notes the cruel irony inherent in having large agri-businesses become alert to the financial resilience benefits of genetic diversity in farming, and the lack of room their business models leave for the caring agrarians who have attentively preserved plant breeding knowledge during the last several decades of productivist agricultural expansion. In the vivid ethnographic accounts of Blanchette (2020) and Gillespie (2016), both human employees and animals experience violent outcomes as a result of the lack of time and space afforded to care in high-tech intensive livestock systems.

In contrast to these pessimistic accounts of technology's relation to care, Chao (2018) notes how the caring instincts of seed breeders and plant nursery workers can flourish *in spite* of the technologically sophisticated agri-business nexus within which they operate. Krzywoszynska (2019a, 2019b) notes the way soil measurement techniques and soil science expertise can help farmers tune in to the needs of the nonhumans on the farm and refine their caring practice. There are, then, potential synergies that exist between agricultural technology, natural scientific methods, empirical measurement, affect, care and the mitigation of multispecies violence (Puig de la Bellacasa 2019). New technologies thus offer up awkward questions about the practical guidance around caring for the more-than-human world on the farm.

## III Agricultural care: *cui bono?*

The problems facing the food system are complex. How to produce enough food to feed a large and growing population without converting more land to agricultural usage? How to challenge the concentration of power in the food system without forgoing the production efficiencies secured through the past 70 years of age-tech development? How to avail of the carbon offsetting and biodiversity potential of soils and trees without exerting pressure on other areas to expand and intensify food production? How to preserve the cultural heritage tied up with livestock husbandry without creating land use competition between food for humans and feed for animals or generating excessive greenhouse gas emissions? How to have robust policies to reduce agro-chemical applications without just shifting input usage to parts of the world with weaker political governance?

These questions aren’t exhaustive of the problems besetting sustainable and peaceable food system design, but they are illustrative of their locally specific implications and globally entangled character. They hint at the manner in which individual farmers and food producers are imbricated in a vast network of relations where decisions about how and what to produce, and where and to whom to sell have the potential to cause violence and/or enact care well beyond the confines of farm gate (Newman et al. 2022; Yao et al. 2018).

If care ethics is being positioned as part of the salve to agriculture's role in driving contemporary socio-ecological crisis (Krzywoszynska 2019a)—an agri-food proposition not just “in” the Anthropocene, but “for” it (Maye et al. 2022)—how can it do so without producing violence elsewhere in the food system? To do so, it needs to be able to see specific food productions contexts in relation to spatial and temporal dynamics of change that are more expansive in character (see Tsing et al. 2019 on the “patchy Anthropocene”). Through its focus on relational ontologies and encounter-based ethics, however, there is a risk that current formulations of agricultural care are better able to engage with the tensions between violence and care in local spaces than in remote ones.

Scholars using agricultural care in their research describe, for example, the way their research participants are attentive to and affected by the more-than-humans on the farm or in its immediate environs. The enactment of care and the reduction of violence is, in these settings, indexed primarily to local outcomes: healthier soils, more affective engagement on the part of the farmers in their environmental milieu, more peaceable human–nature relations and so on. In contrast, food system trends whose changes are slow, distant, accretive or diffuse are much less able to make the same sort of affective claim on participating farmers and researchers. The socio-ecological violence generated by intensive food production and agro-chemical manufacturing and application (Galt 2014; Wolford 2021) is, for example, being (re)distributed by a spatially heterogenous process of agricultural abandonment, expansion and de/intensification (Laurance et al. 2014; Zabel et al. 2019) and an uneven geography of development (Werner et al. 2022). Such trends are driven by a range of political, environmental and economic factors that include, by way of example, the ratcheting up of environmental protections in wealthier parts of the world like the EU that produce system leakages which offshore environmental damage to other (usually poorer) nations (Fuchs et al. 2020). When laboring on and engaging with particular farmed landscapes, however, these slow and remote dynamics of change fail to present themselves as somatic and affecting things. As a result, they surface much less easily as objects of concern in an ethical framework organized around affect, encounter and ontological entanglement.

The implication is not that the scholars using agri-care ethics are not engaged with these issues. It is, to the contrary, clear their work is animated by ecological and social justice concerns. Nor am I implying that the producers and farmers at the heart of their studies are not already care-fully acting on a similar set of global food system priorities. The point, rather, is that the agricultural care framework as it has so far been mobilized lacks some of the conceptual grammar needed to codify ethically significant outcomes that fall outside the bounds of affecting experience, multispecies encounter and local socio-ecological outcomes.

These problems are not new ones for care ethics. Nor are they unique to these agricultural and food production incarnations. Surveying the literature at the intersection of geography, care, and ethics, Popke concludes that “If relations of care are affective, embodied and relational, then an ethics arising out of this would seem to be necessarily partial and situational, holding only for those with whom we have some immediate contact and familiarity” (2006: 507). Tronto's *Moral Boundaries* (1993) is dedicated to tackling the distance-decay problem by transplanting care from a “women's morality” onto a “public ethic of care.” Such a framework would compel us to consider those whose voices are quietest in society, rather than just those who have the means to be heard. She describes the need to develop robust democratic institutions to act as a guard against care's tendencies towards particularism and ghettoization. The implication is that without dedicated efforts, care is partial in its coverage, and it tends to visit a violence on those not selected for its attentions (Martin et al. 2015).

Similar concerns have been directed towards the post-humanist scholarship the agri-care work draws on. Hornborg (2017) worries that object-orientated and relational ontologies have created academic atmosphere problematically unengaged in the political economy of the Anthropocene. In relation to Haraway's (2016) Children of Compost fable depicting a future populated with human–animal hybrids, he asks, “how does it feed its inhabitants?” (2017: 7). These details matter*.* The political economy involved in the production and delivery of food, along with the accumulation of wealth such activities create, is bound to structure the distribution of violence and care visited on the inhabitants of Haraway's fictional world. Hornborg takes the decision to flesh out certain aspects of the world (that its protagonist is part-human, part-Monarch butterfly) and not others (how people are fed) as reflective of a problematic assumption that any world that has got its more-than-human relations sorted out will also champion a fair and equitable political economy. His concern is that that we should not let ourselves assume that a society has fairly apportioned out the food system's violent and salutary implications just because it has embraced progressive notions of more-than-human entanglement and affecting multispecies encounter.

Given the central role afforded to this post-humanist discourse in the agricultural care literature reviewed above, it is unsurprising how these concerns mirror those raised above. In learning to be affected by the complex more-than-human considerations involved in producing food, local socio-ecological outcomes and expressions of ontological entanglement acquire an urgent ethical currency in a way that complex, and accretive political economy considerations do not. Complaints of this nature have (and already *had*) begun to be acted on by scholars writing from the intellectual traditions Hornborg criticizes. Ginn (2014), Giraud et al. (2019), Giraud (2019, 2021), and van Dooren (2011) consider the violence inherent in multispecies entanglements, and the political economy behind the uneven distribution of the socio-environmental harms they create. Giraud et al. (2019) develop the idea of *abundance* to conceptualize the way biodiversity loss and ecological simplification have created conditions for the flourishing of awkward creatures (like bed bugs) that produce harmful outcomes for some of the actants in the entanglements they help form. To ensure that work predicated on relational ethics and more-than-human ontological entanglement does not err into an ahistorical and apolitical analysis that is inattentive to the distribution of these harms, Giraud (2019) shows how the entanglements that are foreclosed on by abiding multispecies arrangements are just as ethically significant as those that have been allowed or made to emerge. These counterfactuals can then be used as a foothold for the development of a more politically engaged relational ontology.

Krzywoszynska (2019a), building on Pitt (2018), gets closest to confronting these issues in the context of agricultural care. She recognizes that the potential of care as an ethico-political project is jeopardized when it is framed in an overly parochial way. Gaps in ethical coverage are especially likely for things that are un-sense-able (too small, too big, too remote) to the care giver. Krzywoszynska (2019a) invokes the idea of *speculation* to bridge the gaps that can open up between sense-ability, attentiveness, affect, and the enactment of agricultural care. Being speculative prompts the care giver to avoid complacency in the enactment of care; to constantly reflect on the needs of the care-receiver; to be experimental in the way one seeks to become affected by the thing being cared for; and to remain modest about the extent to which can ever fully apprehended the needs of the agro-ecosystem in question (Krzywoszynska 2019a).

It is unclear, though, how successful these contributions can be in addressing to the issues outlined above. The theoretical developments Krzywoszynska (2019a) puts forward to navigate the affective and sensory outages that can frustrate care are written in relation to the land managers whose caring commitments risk puttering out of life as their ability to sensorily grasp the complexity of the farm's multispecies assemblage bottoms out. They are not designed to help farmers learn to be affected by remote yet ethically significant outcomes; nor are they written to furnish academics with the theoretical tools they need to evaluate how the enactment of agricultural care can be done for more-than-human actants both within and without the farm. To solve these issues, an updated model of care is needed—albeit one still centered on the valorization of affect, attentiveness and speculation.

## IV Metabolism and material flows

My central claim in this paper is to suggest that care's exposure to particularism can be combatted by directing critical attention towards the material inflows and outflows of the farms and food production environments being studied. These can be understood as a part of their *metabolism*. The term metabolism refers to the ongoing circulations and transformations that continually rearrange humans and nonhumans in new ways (Swyngedouw 2006). It is explicitly material in its conceptual construction, focusing on the stuff that enters, moves around, and leaves a given space, and all those more-than-human inhabitants involved in propelling and shaping those transformations (Newell and Cousins 2015). It is also inherently political, in that it attends to the way that the violent and salutary outcomes produced through some metabolic circulation are indexed to race, gender and class (Swyngedouw and Kaika 2000). While the agricultural care work so far has majored on the more-than-human transformations and interactions that take place *within* particular farms and food production settings, the notion of metabolism can help draw attention to those materials that come in from elsewhere, and those that leave. Doing so can, I believe, help agri-food scholars gain analytical purchase on the outcomes of food production activities which, although they unfold beyond the farm gate, are nevertheless important for the promotion of care and the mitigation of violence in an ethical food system (Morgan 2010).

The metabolic concept has seen great uptake in urban rather than rural studies (Kennedy et al. 2011). Cities, under the metabolism framework are viewed as socio-ecological cyborgs, hybrids of humans, nonhumans, energy and inert materials (Gandy 2005). These various entities work, move around and interact with one another, creating new material arrangements, generating capital, waste, pollution, and goods and services. The farmed environments at the heart of this paper share these cyborg nature culture (Haraway 2003) qualities. As many scholars featured in the above review show, food production is defined by messy more-than-human agricultual assemblages whose relations to one another are central to their ontological composition (Krzywoszynska and Marchesi 2020; Puig de la Bellacasa 2019). The metabolism concept even has its deep historical roots in studies of agricultural science (von Liebig 1840), where it was used to express anxiety over the emergence of long-distance trade, an urbanizing population, and the attendant separation of spaces of production and spaces of consumption.

Although many metabolic circulations and transformations occur within the bounds of specific urban—including urban food production (Shillington 2013)—hybrids, the circles are rarely fully closed (Swyngedouw 2006). Things enter them and leave them. City planners, for this reason, have to grapple with the way clean water flows into a city, and the way wastewater flows out (Decker et al. 2000). Blockages that impede the entry, circulation, transformation and eventual ejection of this water (or any other relevant material) can stagnate and compromise the hygiene and efficiency of the nature culture hybrid in question (Gandy 2004). For this reason, inflows and outflows are central to evaluations of social and ecological impact (Kennedy et al. 2011). They are the material vectors and the causal linkages that enmesh some nature culture hybrid in the wider system it exists in, and so are essential to understanding the way caring relations and violent outcomes are distributed between actors both within a given space and beyond it.

These ideas have clear application in studies of agri-food scholarship. Food production happens via the metabolic transformations and multispecies interactions that take place in particular sites (photosynthesis, soil repair, crop growth, grazing, animal reproduction, harvest, etc.). Such processes have been placed at the center of empirical frame of the agri-care literature reviewed above. Nutrients, energy, and water are also brought onto the farm, embodied in animal feed, synthetic fertilizers, rain, manures, seed, pesticides, diesel, livestock animals, and so on. Things also leave: run-off of excessive fertilizer and pesticide application, livestock carcasses, grain, vegetables, sileage, greenhouse gas emissions.

The specifics of these inflows and outflows have implications with *prima facie* relevance for the enactment of care and the distribution of violence. Did the farm's nitrogen fertilization come from a neighboring farm who had excess manure to sell, or did it come from a plant in Western Russia using the energy intensive Haber-Bosch process? Did the farm produce a small number of expensive, nutrient rich vegetables to be sold directly to local consumers or did it produce vast quantities of grain to be shipped around the world to be processed and included in affordable foodstuffs? Were the livestock carcases being sold produced in modern, emissions-efficient, indoor feedlot systems reliant on the international soya supply chains driving deforestation in the Amazon, or were they produced by grazing the farm's marginal land that couldn’t be used for cultivation? The food system calculations involved in answering these questions implicate ethically charged trade-offs between production efficiencies, animal welfare, food justice, nutrition, and productivity. The point here isn’t to pass normative judgment on these questions, but to highlight that they yield ethically differentiated answers.

These questions also reveal the pliability of the metabolism concept in studying those outcomes both upstream (ingestion, inflow), mid-stream (transformations, circulations, digestions) and downstream (production, pollution, outflow) of the food production sites being studied. A lot more might—and should—be done to bring the literature around metabolisms into conversation with rural and agri-food scholarship. The work Coplen (2018) has done to integrate metabolism with agri-food studies represents, I believe, the correct direction of travel. She puts the notion of metabolism into conversation with different strands of political ecology to reflect on the way that the undertaking of labor and the movement of goods is central to understanding the socioecological relations embodied in the food system. Similar to her work, I use the metabolism concept to emphasize how the things that move between different nodes in the food system are proper parts of the processes and interactions that happen within them; and that by focusing on metabolic material inflows, outflows and transformations, scholars can conceptualize both the distant and local outcomes being produced through the activities of some food production site.

## V Attentiveness, affect, and speculation in a metabolic agricultural care

While the agricultural care literature can be read as an exploration of the more-than-human metabolic transformations that take place within the spatial confines of some food production case study site, the additional focus on material inflows and outflows raises practical questions. How can agri-food scholars accommodate such complex causal connections as they consider the global food system that specific case study sites operate within? And how to create workable guidance for those looking to evaluate whether and how these varied ethical demands are being fulfilled? To answer these questions, I return to and look to expand the notions of speculation, affect, and attentiveness used in the agri-care literature.

The ideas of attentiveness has, so far, been used to articulate the need to be expansive about the constituency of things that merit their caring attention, diligent in the enactment of care to minimize the violent consequences that a care givers’ incomplete comprehension of relevant more-than-human entanglements might otherwise bring about, and alive to the various ways an agro-ecosystem makes its needs known. I argue that this attentiveness should be expanded out to the materials flowing into and out of particular food production environments. They are the vectors linking them to the broader food system they operate with, and so can be used as the starting point for considering the remote violence and/or care being enacted through the farm's activities. This should be paired with attentiveness to the qualitative research, journalism, and modeling work that has sought to make the complex flows of nutrients, agricultural commodities, global land use change, water pollution vivid, and comprehensible. Such work can imbue the materials entering and leaving the farm with the ethical significance of the outcomes they are helping drive elsewhere in the food system. These materials can, as part of this process, then begin to acquire the sort of affective potency that is central to the enactment of care. As Krzywoszynska (2019a, 2019b) shows, the attentiveness achieved through different intellectual, empirical, scientific, somatic and emotive faculties do not have to pull care in different directions; they can act in chorus to broaden its scope and enhance its ethical application. Agri-food scholars might, in this way, learn to be affected by things as seemingly mundane as the animal feed entering the farm or the produce leaving it by being attentive to all those intellectual activities that have made their upstream/downstream impacts knowable.

The spirit of speculation in this metabolic agri-care context is also an important one. Individual researchers do not need to have fully costed and modeled frameworks calculating the remote caring and/or violent outcomes associated with this or that input or output—but they must nevertheless speculate. Being speculative, as it has done elsewhere in the care literature, can help overcome the unavoidable knowledge gaps involved when faced with inscrutable biophysical (like soil repair) or socio-economic (like the globalized trade in agricultural commodities) processes. It can help “[push] beyond what can be directly experienced by individual bodies” (Krzywoszynska 2019a: 664). So long as relevant actors are attentive to and affected by the work being done to make these dynamics known (i.e., to push beyond what can be known through their individual bodies), they can use the material inflows and outflows to speculate about the distant yet ethically significant outcomes associated with the activities taking place within some food production site.

Researchers have, to use one of questions asked above as an example, documented the causal links between the growth in international trade in soya grown for animal feed and land use changes in Brazil and the Amazon (Nepstad et al. 2006; Yao et al. 2018), and soya's role in conjoining planetary to human health (Beacham 2022). A farm's participation in these supply chains has violent yet distantly experienced outcomes: the replacement of small-scale land ownership and indigenous management with ecologically simplified monocultural plantations (Hetherington 2020; Wolford 2021). By being attentive to and affected by a farm's metabolic inflow of feed products, as well as all the research that has sort to illuminate their distant causal connections, scholars can speculate about the violent and/or caring outcomes associated with different approaches to animal husbandry, both within and beyond the farm gate.

Work has also documented the socio-ecological violence being produced through globally heterogenous pressures of agricultural de/intensification, and the system leakages being created by uneven geographies of development and environmental protection (Fuchs et al. 2020; Werner et al. 2022). Areas in the Global North are typically experiencing abandonment (van der Zanden et al. 2017), afforestation and de-intensification (Winkler et al. 2021), while landscapes in the Global South, particularly in the tropics, are being exposed to pressures to intensify and expand production (Kastner et al. 2021; Lenzen et al. 2012; Winkler et al. 2021). To help counter these trends, food system scholars have integrated redistributive global justice factors into their models. They claim that there is a need for countries in the Global North to increase rather than offshore their contributions to global supply chains (both of food and other goods) to reflect the outsize ecological footprint of their consumption habits (Sun et al. 2022). The need to increase food production in temperate countries, particularly in the Global North, increases further still when accounting for the land rendered agriculturally untenable due to climate change (Beltran-Peña et al. 2020).

A related body of work has sought to describe the optimum spatial distribution of food production as a function of agricultural potential and land use opportunity (Heistermann et al. 2006; McDowell et al. 2018) and the agricultural practices that pertain to both environmental and productivity improvements (Adeux et al. 2019; Lechenet et al. 2017). While the agricultural sector is already highly attuned to environmental and productivity win-wins and an “agricultural potential-appropriate land use” calculus (Cusworth and Dodsworth 2021), social scientists need to be attentive to and affected by these literatures, too. Agri-food scholars might study the quantity, type, and quality of the food flowing out specific case study sites, and speculate about its compatibility with efforts to fairly and care-fully apportion out the spatial distribution of agricultural production and all of the potential violence that entails. This attentiveness will allow them to assess whether some farm is challenging or contributing to a potentially harmful and unjust redistribution of the environmental loads associated with agricultural production. There are, of course, caveats that need to be attached to this approach to care. The actions of those managing specific case study sites are delimited by the socio-political landscape they operate within, and their ethical duties to combat macro-scale food system trends (like the offshoring of environmental damage) need to be understood as a function of those structuring factors. These limits notwithstanding, by considering the inflows and outflows of a farm's metabolism, agri-food scholars can begin to see a farm's activities in relation to the caring and/or violent outcomes being produced both near and far to the sites being studied.

## VI Conclusion

The aim of this paper has been to think through the manner in which general concerns about care's violent potential and partial coverage might apply to the particular case of food production and farm management. Although my petition for social scientists to pay greater attention to the metabolic inflows and outflows of particular food production sites is grounded in the particularities of the food system, it resonates with other solutions to care's aversion to distance. In this concluding section, I tie the paper's contributions back into the feminist ethics literature from agri-care project was initially launched.

Efforts to “stretch” (Bartos 2019) care's boundaries to overcome its distance-affect-decay problem fall into two categories (Popke 2006). The first seeks to infuse the quotidian and ethical merits of care into political concepts, policies, and private/public governance mechanisms. Care, in this way, can be used as a robust and universal code against which different interventions can be measured. This model would look to codify the steps used to achieve care and/or the outcomes it produces to evaluate the extent to which some activity enacts care and minimizes violence for remote actors. In the world of food and farming, organic food accreditation schemes allow retailors to guarantee production standards. This then allows customers to minimize the ecological harms of agro-chemical applications in distant farmed landscapes through their purchasing habits.

The second category relates to the efforts that emphasize the ties that link distant actors together. Care, in this model, is an emergent feature of the interdependency of life and extends out to those that cannot be sensed, to those who are far away, and to those who wield minimal affective sway. Notions of civic, diasporic or global citizenship, for example, might be fostered to generate the desired type of caring commitment to a broader cohort of remote yet connected actors (Svenhuijsen 1998). Groups of farmers who share information about how to care for more-than-human agro-ecosystems as a way of expanding their care across a broader geographic range fall into this latter category. This approach to overcoming care's distance-decay problem has better fit with the agri-food literature reviewed above. There, care is messy (not codifiable), context-specific (not universalizable), and the product of ongoing engagements (not discrete governance mechanisms) between different actants in the multispecies assemblage in question (Puig de la Bellacasa 2017).

The difference between these two solutions is manifest in the distinction between the terms caring *about* and caring *for.* Where caring about refers to the emotive connections that shape caring relations and precede everyday caring doings, caring for involves its embodied enactment (Milligan and Wiles 2010). As feminist geographers have shown, certain aspects of care (i.e., about) are less subject to frustration than others (i.e., for) when spatial expanses open up between actors (Grant et al. 2004). It is easier, in other words, to care about someone or something far away (like a distant farmed landscape) than it is to care for them. The trick is to avail of the spatial robustness of the capacity to care *about* when developing guidance for the enactment of caring *for* at distance*.*

Fortunately, the two aspects of care are not fully separable from one another—and spatial proximity is not always a good predictor for evaluating what sort of care is at work in any given scenario. Poorly paid care workers care for others even when they do not necessarily care about them (Yeates 2004), while contact via phone and video calls can allow people to care for their loved ones even at distance (Milligan 2006). Reading across this literature, Milligan and Wiles (2010) conclude that emotional proximity is just as powerful a structuring force in shaping the presence of care as spatial proximity is. To moor Popke's two solutions to the distance-decay-affect problem to one another, and to install the spatially durable qualities of caring *about* into the embodied enactment of caring *for*, Milligan and Wiles (2010) advocate for a model of care that stresses the ethical potency of emotional proximity in instances of spatial distance. In such a framework—what they term the *landscapes of care*—the “values from an individual's personal emotional relationships become applied to more spatially distant social relationships… as a way of thinking ethically and acting responsibly in an increasingly interconnected world” (Milligan and Wiles 2010, 742). An agricultural ethics of care would, under these conditions, encourage intellectual and emotional responsibilities to distant more-than-human actors to be enmeshed with insight about the globally interconnected web of the food system. Attentiveness to the work that has made these causal interconnections affecting would then represent, in this model, meaningful input for those evaluating the ways in which specific management practices distribute care and violence between both local and remote actors.

The ingenuity of the agri-care literature so far has been to enmesh farmers in the more-than-human composition of the farm, and to encourage caring relations to flourish from shared ontological rootstock. To fold this framework in with insight from earth system scientists researching into the food system (Smil 1999; Verburg et al. 2015), those farmers are equally enmeshed in the global flows of emissions, foods, nutrients, water, biodiversity and land use change, linked together by webs of supply chains, trade deals, and consumer habits. By foregrounding the fact that a farm's inflows and outflows are a proper part of its metabolic transformations, I believe these remote causal relations can emerge as affecting ethical input for care's enactment. My submission here falls into the Popke's (2006) second type of solution to care's distance-decay proclivities. By being attentive to material vectors that root a case study into the system it operates within, the food system's global web is brought into sharper focus. These connections usher in a new set of affecting considerations and caring responsibilities. They furnish the agri-care project as it has so far been imagined with the duty to act in the speculative hope that care can flourish, and violence can be minimized across a broader geographic range. It is, for these reasons, an agenda for agri-food research not just “of” but “for” the Anthropocene (Maye et al. 2022).

## Notes

1. Disambiguation—except for this one, all references to Giraud relate to Eva Giraud, not Esteve Giraud.
